# Negative Plant-Soil Feedback Driven by Re-assemblage of the Rhizosphere Microbiome With the Growth of *Panax notoginseng*

**DOI:** 10.3389/fmicb.2019.01597

**Published:** 2019-07-26

**Authors:** Lifen Luo, Cunwu Guo, Luotao Wang, Junxing Zhang, Linmei Deng, Kaifeng Luo, Huichuan Huang, Yixiang Liu, Xinyue Mei, Shusheng Zhu, Min Yang

**Affiliations:** ^1^Key Laboratory for Agro-biodiversity and Pest Control of Ministry of Education, Yunnan Agricultural University, Kunming, China; ^2^State Key Laboratory for Conservation and Utilization of Bio-Resources in Yunnan, Yunnan Agricultural University, Kunming, China

**Keywords:** *Panax notoginseng*, microbiome, soil-borne pathogens, rhizosphere, negative plant-soil feedback

## Abstract

There is a concerted understanding of the accumulation of soil pathogens as the major driving factor of negative plant-soil feedback (NPSF). However, our knowledge of the connection between plant growth, pathogen build-up and soil microbiome assemblage is limited. In this study, significant negative feedback between the soil and sanqi (*Panax notoginseng*) was found, which were caused by the build-up of the soil-borne pathogens *Fusarium oxysporum*, *F. solani*, and *Monographella cucumerina*. Soil microbiome analysis revealed that the rhizospheric fungal and bacterial communities were changed with the growth of sanqi. Deep analysis of the phylum and genus levels corroborated that rhizospheric fungal Ascomycota, including the soil-borne pathogens *F. oxysporum*, *F. solani*, and especially *M. cucumerina*, were significantly enriched with the growth of sanqi. However, the bacteria Firmicutes and Acidobacteria, including the genera *Pseudomonas*, *Bacillus, Acinetobacter* and *Burkholderia*, were significantly suppressed with the growth of sanqi. Using microbial isolation and *in vitro* dual culture tests, we found that most isolates derived from the suppressed bacterial genera showed strong antagonistic ability against the growth of sanqi soil-borne pathogens. Interestingly, inoculation of these suppressed isolates in consecutively cultivated soil could significantly alleviate NPSF. In summary, sanqi growth can suppress antagonistic bacteria through re-assemblage of the rhizosphere microbiome and cause the accumulation of soil-borne pathogens, eventually building negative feedback loops between the soil and plants.

## Introduction

Plant-soil feedback is the phenomenon that plant affects the soil, which in turn affects the growth of the same or other plants (Bever et al., 1997; Ehrenfeld et al., 2005). According to the responses of plants or their offspring, feedbacks can be divided into positive, negative or neutral feedback (van der Putten et al., 2013). Negative plant-soil feedback (NPSF) often reduces plant biomass (van der Putten et al., 2013). Most studies demonstrated that NPSF plays a major role in maintaining plant species diversity in natural systems (Kulmatiski et al., 2008). However, NPSF in agro-systems led to a severe decline in crop productivity (Ogweno and Yu, 2006). In particular, some medicinal crops, such as *Rehmannia glutinosa*, *Pseudostellaria heterophylla*, *Salvia miltiorrhiza*, and *Panax notoginseng*, exhibit strong NPSF (Tan et al., 2012; Yang et al., 2015).

Although nutrient imbalance and the accumulation of autotoxins in rhizosphere soil are considered as factors of NPSF, the changes of rhizosphereic microbes, especially the build-up of soil-borne pathogens, have been proven to be the major driving factors of NPSF (Packer and Clay, 2000; Ogweno and Yu, 2006; Mangan et al., 2010). The increasing evidences showed that the rhizosphere was a key zone for plant-microbe interactions and plant health (Zachow et al., 2014; Tkacz et al., 2015). Plants modify the community and function of the rhizospheric microbes through root exudates (Bais et al., 2006; Chaparro et al., 2014). Correspondingly, the rhizospheric microbes in turn offer a series of services to the plant, such as nutrient acquisition, abiotic tolerance, and diseases or pest resistance (Mendes et al., 2013). Generally, plants and microorganisms interact positively, but many plants show serious negative interactions due to the build-up of pathogens or the suppression of antagonistic microbes such as *Pseudomonas*, *Bacillus*, and *Burkholderia* during their growth, especially when they are continually monocultured (Trotel-Aziz et al., 2008; Li et al., 2014; Gao et al., 2015; Park et al., 2015; Yang et al., 2015; Wu et al., 2016; Tahir et al., 2017). Many studies indicated that rhizospheric microbes of a diverse range of plants such as maize, wheat, *Arabidopsis*, pea, sugar beet, and *Medicago*, changed with the development of the plants (Baudoin et al., 2002; Mougel et al., 2006; Houlden et al., 2008; Micallef et al., 2009). However, how the plant drives the changes of rhizosphere microbe community including soil-borne pathogens and biocontrol microbes with growth and its relationship with the rhizospheric microbe community remained unclear.

Sanqi [*Panax notoginseng* (Burk.) F. H. Chen] is a medicinal plant, and its cultivation is hampered by strong NPSF in continuous monoculture system (Yang et al., 2015; Wei et al., 2018). Many studies have shown that soil-borne pathogens of sanqi is the main cause agent of NPSF (Miao et al., 2006; Yang et al., 2015; Wei et al., 2018). In this study, we used sanqi as a model crop to (1) study the correlation between the build-up of soil-borne pathogens and the dynamics of the rhizospheric microbiome using 16S rRNA and ITS gene tag sequencing and (2) identify the ability of some significantly changed culturable microbes to alleviate NPSF. Based on these experiments, we were able to decipher the mechanism of NPSF mediated by changes in the rhizosphere microbiome and then develop potential biological agents to alleviate NPSF in the sanqi production system.

## Materials and Methods

### Measurement of the Feedback Relationship Between Sanqi and Soil

Natural soil, collected from a pine forest without sanqi cultivation history in June 2016 in Xundian County, Yunnan, China (103.13°E, 25.67°N; altitude of 1960 m), was used to test the feedback relationship between sanqi and soil. The surface of soil (10–15 cm) was removed, and the layer between 15 and 30 cm was collected as natural soil. The natural soil was sieved twice (5 mm and 2 mm mesh) to remove plant residues and then blended with sand in a certain ratio (soil: sand = 4:1) in the laboratory. The mixed soil had the following characteristics: pH 5.01; electrical conductivity 71.9 μS cm^–1^; available phosphate 3.56 mg kg^–1^; available potassium 168.13 mg kg^–1^; alkali-hydrolysable nitrogen 81.20 mg kg^–1^; and organic matter 25000 mg kg^–1^ soil. The soil was transferred to the cells of seedling nursing trays (50-cell per tray, 5.0 cm × 5.0 cm × 8.0 cm per cell). Healthy seeds were collected from mature sanqi plants. Seeds were immersed in 1% sodium hypochlorite for 5 min and washed three times with sterile water and then planted into the cells of the seedling nursing trays (one seed was sown per cell). All trays were arranged in the same greenhouse (25 ± 2°C, 12 h light/12 h dark) in a completely randomized block design and watered weekly over the course of the experiment. After 8 months of growth, the plants were uprooted, and the bulk soil was used to evaluate the feedback relationship between sanqi and soil based on a previous method (Mangan et al., 2010; Wei et al., 2018). Briefly, the bulk soil was divided into two parts. One was steamed at 90°C for 15 min, and the other was not treated. Soil without sanqi cultivation was used as a control treatment. Then, the soil from above treatments was collected and pooled into three seedling nursing trays. The seed germination rate and seedling survival rate were recorded in April and November, respectively. The calculation formulas are as follows:

$$\mathrm{The}\mathrm{seed}\mathrm{germination}\mathrm{rate}\left(\%\right)=100\times \frac{\mathrm{emerged}\,\mathrm{seedlings}}{\mathrm{total}\,\mathrm{seeds}\,\mathrm{in}\,\mathrm{each}\,\mathrm{treatment}}$$

$$\mathrm{The}\mathrm{seedling}\mathrm{survival}\mathrm{rate}\left(\%\right)=100\times \frac{\mathrm{living}\,\mathrm{seedlings}}{\mathrm{total}\,\mathrm{seedlings}\,\mathrm{in}\,\mathrm{each}\,\mathrm{treatment}}$$

Pathogen were isolated from infected sanqi seedlings according to a previous method (Lu et al., 2016). Briefly, fresh roots with symptomatic lesions were washed with tap water and then surface sterilized with 1% sodium hypochlorite for 5 min and finally washed three times with sterile water. The surface sterilized tissue was cut into about 5-mm (in length) pieces. The pieces were placed on potato dextrose agar (PDA) with 100 μg⋅ml^–1^ chloramphenicol. After incubated in dark at 25°C for 7 days, single hyphal tips were transferred to PDA. The isolates were identified through morphological and molecular methods. Morphological identification of the isolates was performed with a light microscope based on the morphological features of the spores (Mao et al., 2014). Then, the strains were further identified through ITS amplification (White et al., 1990). The generated sequences were submitted to GenBank and compared with published gene sequences from the National Center for Biotechnology Information (NCBI) website^1^ using the BLAST algorithm. Neighbor-joining (NJ) trees were constructed with MEGA 7.0 software^2^ to generate maximum composite distance matrices for each sequence according to standard parameters (UPGMA with 1000 bootstraps) (Kumar et al., 2016).

A pathogenicity test was performed on sanqi roots *in vitro*. Healthy 1-year-old roots were washed with tap water and then surface sterilized with 1% sodium hypochlorite for 5 min and finally washed three times with sterile water. A mycelium block (6 mm in diameter) was placed on sanqi roots with the mycelial side facing down on roots that did or did not have a pre-made wound. The inoculated roots were placed on moist filter paper in a glass tray and incubated in the dark at 25 ± 1°C. Twenty-four roots were inoculated for each isolate, with non-colonized agar block as a control. After 10 days of inoculation, pathogens were isolated from every root with symptomatic lesions.

### Effect of Sanqi Growth on the Soil Fungal and Bacterial Communities

#### Experimental Design and Growth Conditions

The abovementioned pine soil was mixed with sand in a certain ratio (soil: sand = 4:1). Then, 100 g of soil was placed in one pot (4.0 cm × 4.0 cm × 8.0 cm). Next, two seedlings were transplanted into each pot, and soil without seedlings was used as the blank control. Each treatment, containing 27 replicates, was maintained at three time points (grown for 30, 60, and 90 days). All treatments were incubated in the same growth chamber (with 25°C in the daytime, 20°C at night, 12 h light/12 h dark). The plants were watered once with 1/2 strength Hoagland’s solution and then with fresh water twice a week.

#### Soil Sampling

The rhizosphere soil was sampled following a procedure used previously for *Arabidopsis* (Lundberg et al., 2012) with some modifications. After transplanting, the plants in 9 pots were harvested at specific time points (30, 60, and 90 days). We removed all root surfaces soil until the remaining aggregates were within 1 mm from the root surface. Roots were placed in a sterile 50 mL tube containing 40 mL 1 × PBS buffer. Tubes were vortexed at maximum speed for 15 s to wash off the rhizosphere soil from the roots. The washing buffer was subjected to centrifugation (12000 × *g* for 10 min), and the collecting precipitate was defined as the rhizosphere soil. The soil remaining in the pots after removal of the plants was sampled as bulk soil. The soil without seedlings was also sampled as no-plant soil. Three biological replicates of all treatments at each time point (27 samples; 3 time points: 30, 60, and 90 days) were collected and stored at −80°C for future use. The bulk soil without or with sanqi growth for 90 days in pots were used to evaluate the feedback relationship between sanqi and soil according to the above method.

### Effects of Sanqi Growth on Culturable Fungi and Bacteria in the Soil

The effect of sanqi growth on soil bacteria and fungi was assessed on plates according to Yang et al.’s (2019) method. Briefly, 10 g of each soil sample was added to 90 mL of a 0.1% (w/v) solution of sodium pyrophosphate. The soil suspension was homogenized for 15 min, then decimally diluted (10^–1^ to 10^–7^), and 0.1 mL solutions was plated on nutrient agar (NA) medium containing 15 g peptone, 5 g NaCl, 3 g beef extract, 15 g agar, 1000 mL distilled water, pH 7 (Tchakounté et al., 2018) and rose bengal medium (RBM) containing 5 g peptone, 10 g glucose, 1 g KH_2_PO_4_, 0.5 g⋅MgSO_4_⋅7H_2_O, 15 agar, 100 mL 1/3000 rose bengal solution, 1000 mL distilled water, 0.1 g Chloramphenicol (Zhou et al., 2014) for bacteria and fungi, respectively. After incubating at 25°C for 4 to 5 days, the colony forming units (CFUs) were counted, and mean values were obtained from counts of four replicates. The results were expressed as CFU per gram of dry soil. The isolation was repeated three times in the laboratory. Individual colonies from rhizosphere soil were picked out and inoculated on NA media (bacteria) or PDA (fungi) to obtain cultures, which were then stored at 4°C.

### Sequence Analysis of the ITS and 16S rRNA Genes

Total DNA was extracted from a total of 0.5 g of soils. Extractions were carried out using the Power Soil^®^ DNA Isolation Kit (MO BIO Laboratories, Inc., Carlsbad, CA, United States) according to the manufacturer’s instructions. DNA quality was assessed using a NanoDropTM 2000 spectrophotometer (Thermo Fisher Scientific, United States). Purified DNA was stored at −80°C for future use.

Fungal ITS and bacterial 16S rRNA genes in the rhizosphere soil total DNA samples were sequenced using the Illumina MiSeq platform of Novogene Corporation (Beijing, China). The fungal genes were amplified with the primer sets ITS5-1737F/ITS2-2043R (ITS1 gene region) (Huang et al., 2016), and bacterial genes were amplified with the primer sets 515F/907R (16S rDNA V4-V5 gene region) (Ye et al., 2016). Sequences were spliced using FLASH^3^ (FLASH V1.2.7) (Magoč and Steven, 2011) and were quality filtered (Bokulich et al., 2012). Sequenced reads were assembled for each sample based on the unique barcode using QIIME^4^ (Caporaso et al., 2010). Through quality filtering and chimera removal^5^, the retained effective tags were used to perform operational taxonomic unit (OTU) clustering and species annotation. OTUs were defined at ≥97% sequence identity using UPARSE software (UPARSE v7.0.1001)^6^ (Edgar, 2013). The taxonomic identities of isolates were determined using RDP software (Wang et al., 2007) and Silva schemes^7^ (Quast et al., 2013). Finally, the data of each sample were processed by normalization based on the minimum data in the sample, and then community richness and diversity analysis and principal coordinate analysis (PCoA) were conducted.

All sequences of ITS and 16S rRNA genes can be found in the Short Read Archive (SAR) at NCBI^8^ under accession number PRJNA529039.

### Isolation and Identification of Antagonistic Microbes

The abovementioned culturable microbes from rhizosphere soil were screened on the basis of their antagonistic activity against pathogens isolated from infected plants in a dual culture following the method described in a previous study with some modifications (Sun et al., 2018). A pathogen mycelium block (6 mm diameter) was placed in the middle of the Petri dish. Then, the four culturable microbes were placed at the same distance (25 mm) around the pathogen mycelium block. Plates with only pathogens grown on PDA were used as controls. Four replicate plates were used per treatment. All treatments were incubated at 25°C for 5 days. The mycelium growth of the pathogen was determined by measuring the colony semidiameter. The growth inhibition rate was calculated as follows:

$$\;\mathrm{Growth}\mathrm{inhibition}\mathrm{rate}\left(\%\right)=100\times \frac{\left(\mathrm{radial}\,\mathrm{growth}\,\mathrm{of}\,\mathrm{control}-\mathrm{radial}\,\mathrm{growth}\,\mathrm{of}\,\mathrm{treated}\,\mathrm{sample}\right)}{\mathrm{radial}\,\mathrm{growth}\,\mathrm{of}\,\mathrm{control}}$$

The ability of culturable bacteria to solubilize phosphorus (Nautiyal, 1999), dissolve potassium (Meng et al., 2012) and fix nitrogen (Gao et al., 2015) was also evaluated as previously described. The antagonistic fungi were identified through a morphological method and ITS amplification. The antagonistic bacteria were molecularly identified through 16S rDNA amplification (Cai et al., 2012).

### Evaluation of the Ability of Antagonistic Isolates to Alleviate NPSF

A pot experiment was conducted to assess the ability of selected isolates (inhibition percentage ≥30%) to alleviate NPSF in consecutively cultivated soil. Soil was collected at a 20 cm depth at harvest from sanqi fields at the experimental station of Yunnan Agricultural University, Xundian County, Yunnan, China (103.13°E, 25.67°N; altitude of 1880 m), where sanqi was cultivated for one consecutive year from 2015 to 2016. The consecutively cultivated soil was placed in pots (4.0 cm × 4.0 cm × 8.0 cm per pot), and ten surface-sterilized seeds were sown in each pot and then inoculated with 30 mL (10^6^ cfu⋅mL^–1^) of antagonistic isolate suspension. Pots without inoculation were used as the blank control. The pots were placed in a growth chamber with L/D cycle of 16 h /8 h at 25°C. Each treatment contained three replicates, and every replication included six pots. Three months after treatment, the seed germination rate and plant fresh biomass were recorded.

### Statistical Analysis

Data were analyzed using SPSS version 17.0 software (SPSS Inc., Chicago, IL, United States). Normality of distribution and homogeneity of variance were checked before statistical analysis. One-way analysis of variance (ANOVA) and Duncan’s multiple range test (*P* < 0.05) were used to analyze the mean separation among treatments.

## Results

### Sanqi Has Obvious NPSF Due to Root-Rot Pathogen Infection

**FIGURE 1:**
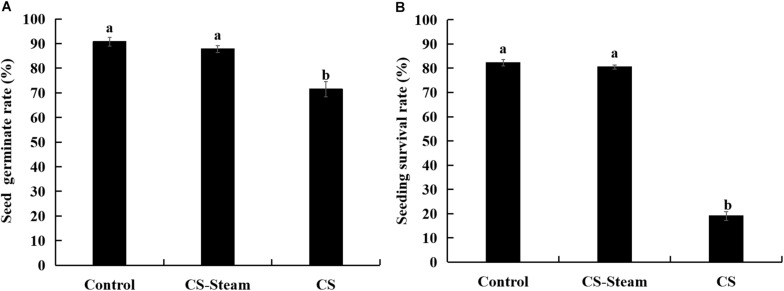
Seed germination **(A)** and seedling survival rate **(B)** of sanqi in continuously cultivated soil and uncultivated soil. Control represents the uncultivated sanqi soil, CS indicates the continuously cultivated soil with sanqi, CS-steam indicates the CS was treated with steam at 90°C for 15 min. The values represent the means ± SE. Different letters on the bars indicate significant differences between different treatments (*p* < 0.05; *n* = 3).

The germination rates significantly reduced when sanqi seedlings were replanted in the continuously cultivated soil (Figure 1A). As the sanqi seedlings grew, their survival rates significantly decreased (Figure 1B). When the soil was sterilized with steam at 90°C for 15 min, the seed germination and seedling survival rates were restored to the level of the control treatment (Figures 1A,B).

**FIGURE 2:**
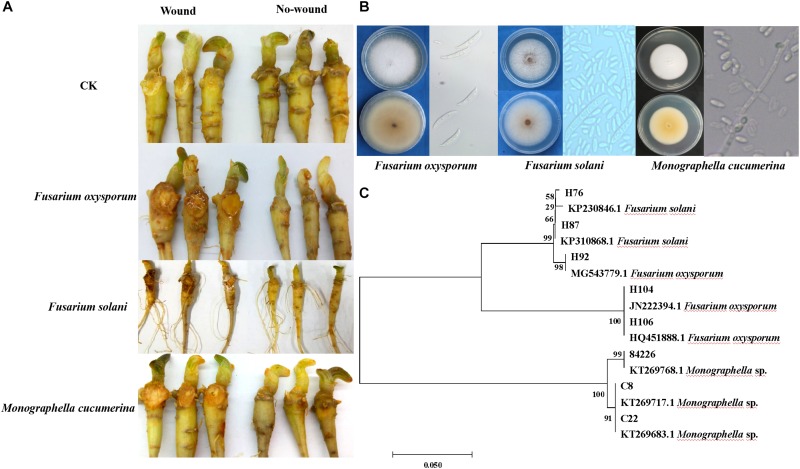
Identification of pathogens causing sanqi rot root and pathogenicity tests. Pathogenicity tests were performed on sanqi roots *in vitro* **(A)**. Colony morphology and conidial characteristics of *Fusarium* spp. and *Monographella cucumerina* **(B)**. Hierarchical clustering of ITS genes of pathogens **(C)**.

A total of fifty fungi were isolated from dead plants with the symptom of root-rot. Among them, eight isolates showed pathogenicity to sanqi roots. Based on colony morphology, conidial characteristics and ITS sequences, three isolates of *Fusarium oxysporum*, two isolates of *F. solani*, and three isolates of *Monographella cucumerina* were identified (Figures 2B,C). For *F. oxysporum* and *F. solani* isolates, root-rot symptoms were typical. *M. cucumerina* isolates showed weak pathogenicity. For most isolates on non-punctured roots, no symptoms were observed (Figure 2A). The control roots did not show any symptom of root rot, and no *M. cucumerina* and *Fusarium* spp. isolates were obtained.

### Sanqi Growth Affects the Soil Fungal and Bacterial Communities

There was NPSF in the soil cultivated with sanqi for 90 days (Supplementary Figure S1). The number of soil microbes quantified on plates demonstrated that sanqi growth had a greater effect on the number of culturable microbes in rhizosphere soil than in bulk soil, and the number of bulk soil microbes was not significantly different compared with no-plant soil (Supplementary Figure S2). Compared to no-plant soil, the fungal population in rhizosphere soil was promoted at 30 days and then suppressed. The bacterial population was significantly suppressed with sanqi growth (Supplementary Figure S2). Thus, the ratio of fungi to bacteria was decreased at 60 days and then increased (Supplementary Figure S2).

**FIGURE 3:**
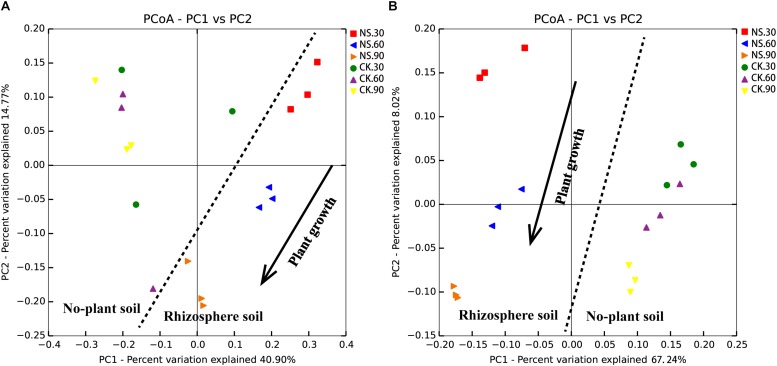
PCoA of no-plant soil and rhizosphere fungal **(A)** and bacterial **(B)** communities associated with sanqi grown for 30, 60 and 90 days, based on the Bray distance metric. NS30, NS60, and NS90 indicate rhizosphere soil from sanqi grown for 30, 60, and 90 days, respectively; CK30, CK60, and CK90 indicate no-plant soil collected at 30, 60, and 90 days, respectively.

Subsequently, the rhizosphere soil samples and their corresponding no-plant soil control samples were analyzed by MiSeq sequencing. A total of 17877 fungal OTUs and 59562 bacterial OTUs were obtained to analyze the changes in the fungal and bacterial communities (Supplementary Tables S1, S2). The PCoA results indicated that the communities of the rhizosphere fungi and bacteria were significantly differed from that of the no-plant soil communities at 30, 60, and 90 days (Figures 3A,B). Both fungal and bacterial communities gradually separated with the growth of sanqi over time (Figures 3A,B).

**FIGURE 4:**
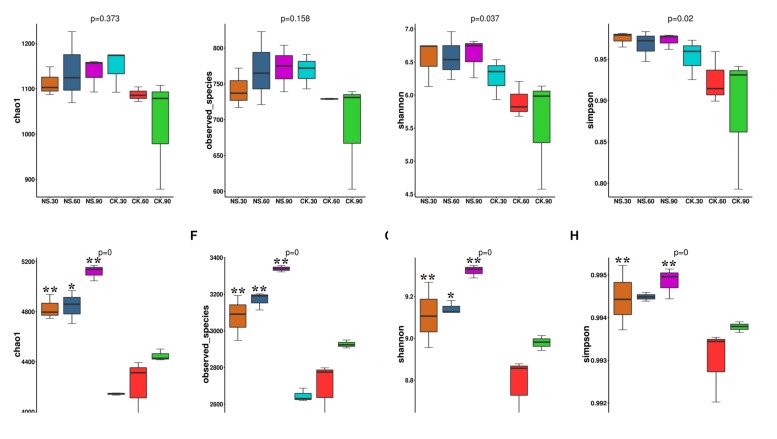
The community richness and diversity indexes of the fungal community **(A–D)** and bacterial community **(E–H)** in both rhizosphere soil and no-plant soil evaluated by MiSeq sequencing. NS30, NS60, and NS90 indicate rhizosphere soil from sanqi grown for 30, 60, and 90 days, respectively; CK30, CK60 and CK90 indicate no-plant soil collected at 30, 60, and 90 days, respectively. The values represent the means ± SE. An asterisk (^∗^) indicates that the differences between sanqi and its corresponding control treatment at the same time were significant at *p* < 0.05. An asterisk (^∗∗^) indicates that the differences were significant at *p* < 0.01.

Although the richness and diversity of fungal community did not show significant differences among the time points, Simpson and Shannon indexes in rhizosphere soil were high compared with their corresponding no-plant soil control samples at 60 and 90 days (Figures 4A–D). Compared to no-plant soil, bacterial richness (Chao1 and Observed-species) (Figures 4E,F) and diversity (Simpson and Shannon indexes) (Figures 4G,H) were significantly increased in rhizosphere soil.

### Sanqi Growth Suppressed Beneficial Microbes but Enriched Soil-Borne Pathogens

Sanqi growth changed the communities of fungal and bacterial at the phylum level (Supplementary Figures S3, S4). With respect to rhizospheric fungi, the relative abundance of Ascomycota was significantly suppressed (*p* < 0.05) by sanqi compared with the no-plant soil at 30 and 60 days but increased compared to the no-plant level at 90 days (Supplementary Figure S3a). In contrast, the abundance of Basidiomycota was significantly increased (*p* < 0.05) at 30 and 60 days but decreased at 90 days (Supplementary Figure S3b). The abundance of Zygomycota was significantly increased by sanqi at 90 days (Supplementary Figure S3c).

With respect to rhizobacterial taxa, ten phyla changed significantly (Supplementary Figure S4). Compared with no-plant soil, the relative abundances of Gemmatimonadetes, Actinobacteria, Planctomycetes, Chloroflexi, Firmicutes and Acidobacteria were significantly suppressed (Supplementary Figures S4a–f), but the relative abundances of OD1, Bacteroidetes, Verrucomicrobia and Proteobacteria were significantly increased (Supplementary Figures S4g–j). With the growth of sanqi, the relative abundances of Firmicutes and Acidobacteria were significantly decreased (Supplementary Figures S4e,f), but the relative abundances of Gemmatimonadetes, Actinobacteria and Chloroflexi were significantly increased (Supplementary Figures S4a,b,d).

**FIGURE 5:**
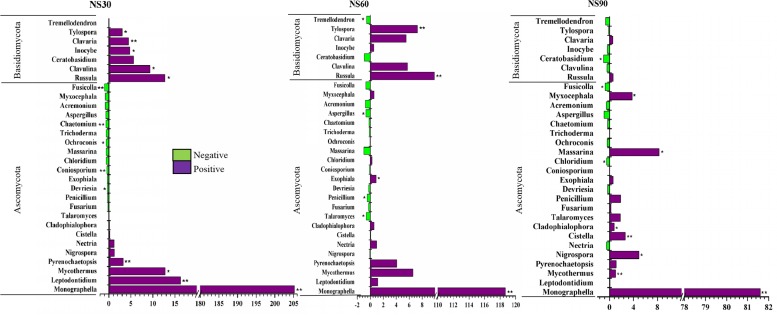
Comparison of fungi abundance differences between rhizosphere and no-plant soil at the genus level. The values represent the means ± SE. An asterisk (^∗^) indicates that the differences in fungi abundance between sanqi and its corresponding control treatment at the same time were significant at *p* < 0.05. An asterisk (^∗∗^) indicates that the differences were significant at *p* < 0.01. NS30, NS60, and NS90 indicate rhizosphere soil from sanqi grown for 30, 60, and 90 days, respectively.

Further analysis demonstrated that twenty-three fungal genera changed significantly at 30, 60, and 90 days. Among them, seven genera belong to Basidiomycota, which increased in abundance in response to plant growth at 30 days (Figure 5). Eight genera belonging to Ascomycota were enriched in relative abundance with the growth of sanqi; however, fifteen genera were suppressed with the growth of sanqi (Figure 5). Among them, the abundance of *Trichoderma*, a typical biocontrol agent, was suppressed with sanqi growth at 30, 60, and 90 days. However, the abundance of *Fusarium* and *Nectria*, soil-borne pathogens of sanqi, gradually increased with the growth of sanqi (Figure 5). It is worth mentioning that the abundance of the pathogen *Monographella* was significantly increased in rhizosphere soil with the growth of sanqi (Figure 5).

**FIGURE 6:**
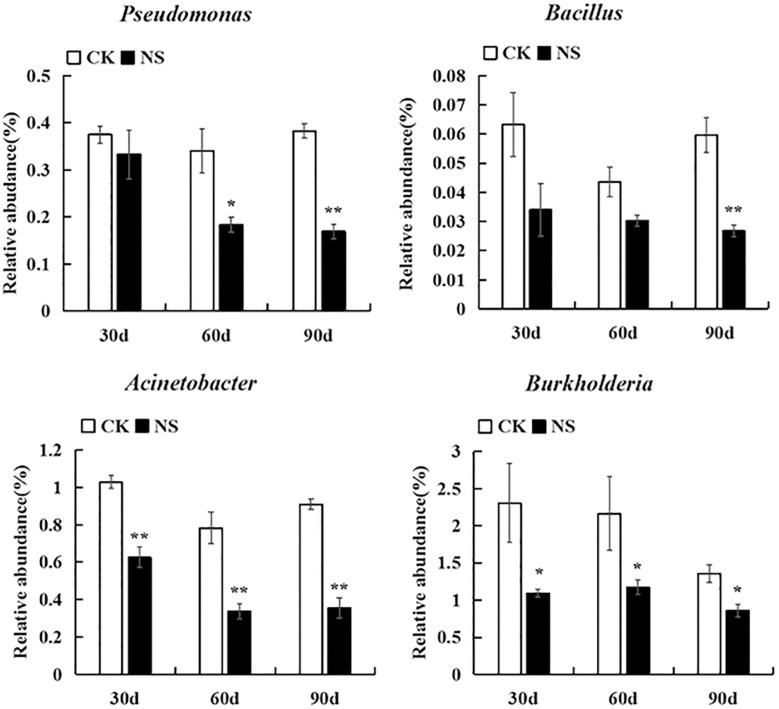
Bacteria with potential biocontrol were suppressed. The values represent the means ± SE. An asterisk (^∗^) indicates that the differences between sanqi and its corresponding control treatment at the same time were significant at *p* < 0.05. An asterisk (^∗∗^) indicates that the differences were significant at *p* < 0.01. CK represents no-plant soil. NS represents rhizosphere soil from sanqi.

With respect to rhizosphere bacteria, a total of ninety-six genera were significantly changed; they belonged to Acidobacteria, Actinobacteria, Firmicutes, Planctomycetes, Bacteroidetes, Proteobacteria, and Verrucomicrobia. Among these genera, thirty genera were suppressed, and sixty-six genera were enriched compared with no-plant soil (Supplementary Figures S5–S11). Some genera related to plant growth, such as *Labrys, Mesorhizobium*, *Bradyrhizobium* and the denitrifying bacteria *Azohydromonas*, were significantly increased (Supplementary Figure S10). Some genera with potential biological control functions, such as *Pseudomonas*, *Bacillus*, *Acinetobacter*, and *Burkholderia*, were suppressed (Figure 6).

### Suppressed Beneficial Microbes Showed Antagonistic Activity Against Soil-Borne Pathogens

**FIGURE 7:**
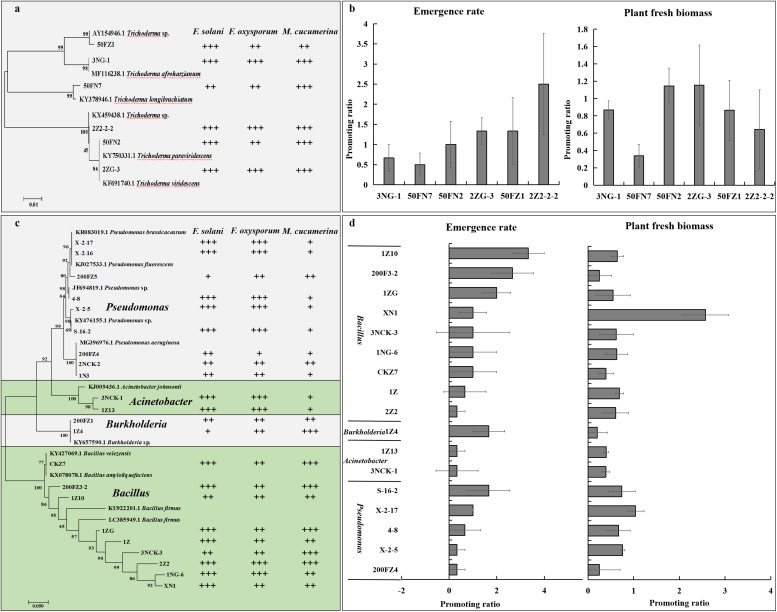
Effect of beneficial fungi and bacteria on the growth of sanqi and pathogen. Hierarchical clustering of ITS genes of *Trichoderma* spp. isolated from the rhizosphere soil of sanqi and their antagonistic activity against *F. oxysporum*, *F. solani*, and *M. cucumerina* **(a)** and the effect of antagonistic *Trichoderma* spp. on the seedling emergence rate and fresh biomass of sanqi in consecutively cultivated soil **(b)**. Hierarchical clustering of 16S rDNA genes of bacteria isolated from the rhizosphere soil of sanqi and their antagonistic activity against *F. oxysporum*, *F. solani*, and *M. cucumerina* **(c)** and the effect of antagonistic bacteria on seedling emergence rate and fresh biomass of sanqi in consecutively cultivated soil **(d)**. +, ++ and +++ represent the levels of antagonistic activity of the strains against *F. oxysporum*, *F. solani*, and *M. cucumerina*. + indicates that the antimicrobial rate ranges from 0 to 30%; ++ indicates that the antimicrobial rate ranges from 30 to 60%; and +++ indicates that the antimicrobial rate is >60%. $\mathrm{Promoting}\,\mathrm{ratio}=\frac{\mathrm{Treatment}-\mathrm{Control}}{\mathrm{Control}}$. Each bar represents the difference in the emergence rate or fresh biomass between treatments and control.

To determine whether antagonistic fungi could promote plant growth and alleviate NPSF, we isolated six *Trichoderma* spp., including *T. tomentosum*, *T. afroharzianum*, *T. longibrachiatum*, *T. hispanicum*, *T. paraviridescens*, and *T. viridescens* (Figure 7a), then tested their antagonistic activity against the mycelial growth of *M. cucumerina*, *F. oxysporum*, and *F. solani*. The results demonstrated that isolates belonging to different species showed antagonistic activity (Figure 7a and Supplementary Table S3). Simultaneously, six isolates were selected to test their ability to alleviate NPSF in a pot experiment. The results showed that the isolates inoculated into consecutively cultivated soil could promote seedling emergence and accumulation of plant fresh biomass compared with the control treatment (Figure 7b). These data suggested that the inoculation of antagonistic *Trichoderma* spp. in consecutively cultivated soil could alleviate NPSF.

To determine whether antagonistic bacteria could promote plant growth and alleviate NPSF, we isolated 115 bacteria and screened their antagonistic activity against the pathogens *F. oxysporum*, *F. solani*, and *M. cucumerina*. Among these bacteria, 22 isolates showed strong antagonistic activity (Figure 7c and Supplementary Table S4). Nine isolates belonged to *Pseudomonas*, two isolates belonged to *Acinetobacter*, two isolates belonged to *Burkholderia*, and nine isolates belonged to *Bacillus* (Figure 7c and Supplementary Table S4). These strains also exhibited nutrient fixing and activation ability (Supplementary Table S5). Seventeen isolates with antagonistic activity were further selected to test their ability to alleviate NPSF in a pot experiment. The results showed that most isolates could promote seed emergence and the accumulation of plant fresh biomass (Figure 7d). These results revealed that the inoculation of antagonistic bacteria in consecutively cultivated soil could alleviate NPSF.

## Discussion

Negative plant-soil feedback, caused by soil-borne pathogen accumulation, often led to a severe decline in crop productivity (Ogweno and Yu, 2006). Here, we found that the rhizospheric soil microbial community and function were altered with the growth of sanqi. As a result, some beneficial microbes with the ability to inhibit pathogen growth were suppressed. Subsequently, the host-specific pathogens accumulated significantly in the rhizosphere soil. These eventually resulted in the negative feedback between sanqi and soil.

### The Soil Microbial Community Changed With the Growth of Sanqi

Many researchers found that the soil microbial community is greatly influenced by plant species and growth (Aira et al., 2010; Peiffer et al., 2013; Chaparro et al., 2014). In this study, we found dramatic changes in the structure of the fungi and bacteria associated with the sanqi rhizosphere compared to no-plant soil. The number of soil microbes counted on plates showed that the culturable fungi in rhizosphere soil was promoted compared with no-plant soil, whereas the bacterial population was significantly suppressed. It is well known that plants have a directional selection ability such that rhizospheric microbes are different from the microbes found in bulk soils (Bulgarelli et al., 2013; Reinhold-Hurek et al., 2015). Further MiSeq sequencing data revealed that compared to no-plant soil, bacterial richness and diversity increased in rhizosphere soil. These data are in accordance with previous studies showing that the richness, diversity, and relative abundance of taxa in the rhizosphere were different from adjacent bulk soil (Haichar et al., 2008; Peiffer et al., 2013). PCoA confirmed that the fungal and bacterial communities gradually separated with the growth of sanqi and significantly differed from no-plant soil communities (Figures 3A,B).

Further analysis of the fungal communities at phylum level demonstrated that Ascomycota, Basidiomycota, and Zygomycota were the dominant taxa (Supplementary Figure S3). This data is in agreement with previous reports, as Ascomycota, Basidiomycota and Zygomycota were enriched in rhizosphere soil fungal communities of two-year-old sanqi (Miao et al., 2016). With the growth of sanqi, the relative abundance of Ascomycota was significantly enriched, but the abundance of Basidiomycota was significantly suppressed. With respect to rhizospheric bacteria, a core microbiome was established in rhizosphere microbial communities after sanqi was planted, and these bacteria comprising Actinobacteria, Acidobacteria, Planctomycetes, Chloroflexi, Firmicutes, Gemmatimonadetes, Bacteroidetes, Proteobacteria, OD1 and Verrucomicrobia. Among them, Actinobacteria, Acidobacteria, Planctomycetes, Chloroflexi, Firmicutes, and Gemmatimonadetes were suppressed compared with no-plant soil (Supplementary Figure S4). Previous studies demonstrated that *Bacillus* (Firmicutes) (Tahir et al., 2017) and Actinobacteria isolates (Kortemaa et al., 1997; Loqman et al., 2009) are biocontrol bacteria that have been widely used to protect crops from disease, and some Acidobacteria isolates have cellulose decomposition ability (Eichorst et al., 2011), photosynthetic ability (Bryant et al., 2007), and may be involved in the iron cycle (Coates et al., 1999). Sanqi growth suppressed the abundance of Acidobacteria and may cause functional changes related to soil health. Additionally, OD1, Verrucomicrobia, Bacteroidetes, and Proteobacteria were significantly promoted compared with no-plant soil (Supplementary Figure S4). These results suggest that sanqi can select specific taxa of microbes for its growth.

### Sanqi Inhibited Beneficial Microbes but Enriched Pathogen

Numerous previous studies indicated significant negative feedback between the soil and sanqi plants (Yang, et al., 2015; Dong et al., 2016; Wei et al., 2018). Our study further confirmed this phenomenon (Figure 1). There is also accumulating evidence that biotic interactions occurring belowground may play a significant role in determining NPSF (van der Heijden et al., 1998; Packer and Clay, 2000). In this study, high temperatures treatment of soil could completely eliminate the NPSF, indicating that biological agents were key factors for NPSF. Pathogens, including *F. oxysporum*, *F. solani*, and *M. cucumerina*, isolated from dead plants in unsterilized consecutively cultivated soil corroborated the pathogens causing NPSF. These data are in accordance with previous work showing that root rot of *P. notoginseng* is mainly caused by individual or mixed infections of pathogens (Miao et al., 2006; Ni et al., 2011). In this study, *M. cucumerina* (current name: *Plectosphaerella cucumerina*, Arx, 1984) was isolated from dead plants and identified as a sanqi soil-borne pathogen (Figure 2A). *Monographella* species are important plant pathogens that have been reported to infect rice, maize and *Opuntia* (Arx, 1987; Hernández-Restrepo et al., 2016), but this is the first report of *M. cucumerina* as a pathogen of *P. notoginseng*.

Negative plant-soil feedback is caused by many factors, but unbalance of soil microbial community is thought to be the main driving factor (Bulgarelli et al., 2013; Manici et al., 2013). In this study, we found that some genera with potential biocontrol ability, such as *Trichoderma* (Huang et al., 2011), *Pseudomonas* (Trotel-Aziz et al., 2008; Park et al., 2015), *Bacillus* (Trotel-Aziz et al., 2008; Tahir et al., 2017), *Acinetobacter* (Trotel-Aziz et al., 2008) and *Burkholderia* (Gao et al., 2015), were suppressed (Figure 6), but pathogens, such as *Fusarium* and *Monographella*, accumulated in rhizosphere soil (Figure 5). *Fusarium* spp. and *Monographella* spp. are widespread soil microbes (Roncero et al., 2003; Hernández-Restrepo et al., 2016). In this study, our MiSeq sequencing data identified that these two genera of pathogens existed in natural soil without sanqi cultivation history. When sanqi was cultivated, the abundance of these pathogens was enriched. Previous research has also shown that plant growth-promoting bacteria, including *Pseudomonas, Burkholderia* and *Bacillus*, decreased in rhizospheric soil of *P. ginseng* (Li et al., 2014). Our pot experiment, which showed that inoculation of these suppressed isolates in continuously cultivated soil could significantly alleviate NPSF, corroborated that the change in the soil microbiome resulted in NPSF.

Previous work revealed that interactions among microbes play an important role in community dynamics or assembly (Niu et al., 2017). Here, we found that the build-up of soil-borne pathogens may be due to the interaction between pathogens and antagonistic microbes. In *in vitro* dual culture tests, most of the suppressed bacteria belong to the genera *Pseudomonas*, *Bacillus*, *Acinetobacter* and *Burkholderia*, and *Trichoderma* spp. have shown antagonistic activity against pathogens. In a pot experiment, inoculation with these isolates in continuously cultivated soil revealed that these suppressed bacteria and fungi could increase the sanqi emergence rate and plant fresh biomass and then alleviate the NPSF. Previous studies have shown that *Trichoderma*, *Bacillus*, and *Pseudomonas* strains act as effective antagonists against ginseng pathogens, such as *F. oxysporum* and *F*. cf. *incarnatum*, and alleviated the replanting problem (Song et al., 2014; Dong et al., 2018). These data imply that these biocontrol agents can alleviate NPSF and that the build-up of pathogens may be due to the suppression of biocontrol bacteria.

In addition to microbial interactions in communities, root exudates are also the main driving factor in community dynamics or assembly (Bais et al., 2006; Reinhold-Hurek et al., 2015). The difference in root secretion composition and content influences the community structure and function of rhizosphere microorganisms (Klironomos, 2002; East, 2013). The change in the rhizospheric microbiome may be partly driven by root exudates of sanqi. Previous studies have shown that ginsenosides, secreted by *P. notoginseng* and *P. quinquefolius* (Nicol et al., 2003; Yang et al., 2015), could inhibit the growth of *T. hamatum* (Nicol et al., 2003) but stimulate the growth of pathogens, such as *Phytophthora cactorum*, *F. solani*, and *F. oxysporum* (Nicol et al., 2003; Yang et al., 2015, 2018). Therefore, the build-up of soil-borne pathogens may be partly mediated by the secretion of some specific components from the root exudates of sanqi. However, the relationship between the dynamics of sanqi rhizospheric bacteria and root exudates will be future studied.

## Conclusion

The negative feedback of sanqi and soil was caused by changes in the rhizosphere microbial community, especially by the build-up of soil-borne pathogens and the suppression of antagonist microbes. Interestingly, these downregulated microbes, regularly inoculated in the consecutively cultivated soil, could significantly alleviate the negative feedback of sanqi and soil. Although the underlying mechanisms for this process are unclear, there is potential that the application of exogenous potential biological agents can mitigate the negative feedback process in agricultural production.

## Author Contributions

SZ and MY conceived the study and directed the project. LL and CG performed microbial isolation. LL, CG, and LW performed *in vitro* dual culture tests. JZ, LD, and KL performed plant growth promotion test. HH, YL, and XM performed MiSeq sequencing, assembly, and analyses. SZ, MY, CG, and LL wrote the manuscript.

## Conflict of Interest Statement

The authors declare that the research was conducted in the absence of any commercial or financial relationships that could be construed as a potential conflict of interest.

## Footnotes

1 https://blast.ncbi.nlm.nih.gov/

2 http://www.megasoftware.net/

3 http://ccb.jhu.edu/software/FLASH/

4 http://qiime.org/scripts/split_libraries_fastq.html

5 http://www.drive5.com/usearch/manual/chimera_formation.html

6 http://drive5.com/uparse/

7 http://www.arb-silva.de/

8 https://trace.ncbi.nlm.nih.gov/Traces/sra

## Supplementary Material

FIGURE S1 Seed germination **(a)** and plant fresh biomass **(b)** of per sanqi in the bulk soil without or with sanqi growth for 90 days in pots. Control represents the bulk soil without sanqi growth, seedlings represents the bulk soil with sanqi growth for 90 days in pots. The values represent the means ± SE. An asterisk (^∗^) indicates significant differences between different treatments (*p* < 0.05; *n* = 5).

FIGURE S2 The influence of seedlings on culturable fungi **(a)** and bacteria **(b)** and the fungal to bacterial ratio (F/B) **(c)** in the rhizosphere and bulk soil. Control represents colony quantities or the ratio of fungi to bacteria in the soil without seedlings. Bulk soil represents colony quantities or the ratio of fungi to bacteria in the soil after removed seedlings. The values represent the means ± SE. Data in the homochromatic column with different lowercase letters indicate significant differences between different treatment durations (*p* < 0.05; *n* = 3).

FIGURE S3 Effect of *Panax notoginseng* seedlings on the relative abundance of Ascomycota **(a)**, Basidiomycota **(b)** and Zygomycota **(c)**. The values represent the means ± SE. An asterisk (^∗^) indicates that the differences between seedlings and their corresponding control treatment at the same time were significant at *p* < 0.05. An asterisk (^∗∗^) indicates that the differences between seedlings and their corresponding control treatment at the same time were significant at *p* < 0.01. CK represents no-plant soil. Seedlings represents rhizosphere soil from sanqi.

FIGURE S4 Effect of *Panax notoginseng* seedlings on the relative abundance of the dominant bacteria at the phylum level. **a–j** indicate that relative abundances of Gemmatimonadtes **(a)**, Actinobacteria **(b)**, Planctomycetes **(c)**, Chloroflexi **(d)**, Firmicutes **(e)** and Acidobacteria **(f)** were reduced and abundances of OD1 **(g)**, Bacteroidetes **(h)**, Verrucomicrobia **(i)** and Proteobacteria **(j)** were increased after planting sanqi, respectively. The values represent the means ± SE. An asterisk (^∗^) indicates that the differences between sanqi and its corresponding control treatment at the same time were significant at *p* < 0.05. An asterisk (^∗∗^) indicates that the differences were significant at *p* < 0.01. CK represents no-plant soil. Notoginseng seedling represents rhizosphere soil from sanqi.

FIGURE S5 Comparison of Acidobacteria abundance differences between rhizosphere and no-plant soil at the genus level. The values represent the means ± SE. An asterisk (^∗^) indicates that the differences between sanqi and its corresponding control treatment at the same time were significant at *p* < 0.05. An asterisk (^∗∗^) indicates that the differences were significant at *p* < 0.01. CK represents no-plant soil. Seedlings represents rhizosphere soil from sanqi.

FIGURE S6 Comparison of Actinobacteria abundance differences between rhizosphere and no-plant soil at the genus level. The values represent the means ± SE. An asterisk (^∗^) indicates that the differences between sanqi and its corresponding control treatment at the same time were significant at *p* < 0.05. An asterisk (^∗∗^) indicates that the differences were significant at *p* < 0.01. CK represents no-plant soil. Seedlings represents rhizosphere soil from sanqi.

FIGURE S7 Comparison of Firmicutes abundance differences between rhizosphere and no-plant soil at the genus level. The values represent the means ± SE. An asterisk (^∗^) indicates that the differences between sanqi and its corresponding control treatment at the same time were significant at *p* < 0.05. An asterisk (^∗∗^) indicates that the differences were significant at *p* < 0.01. CK represents no-plant soil. Seedlings represents rhizosphere soil from sanqi.

FIGURE S8 Comparison of Planctomycetes abundance differences between rhizosphere and no-plant soil at the genus level. The values represent the means ± SE. An asterisk (^∗^) indicates that the differences between sanqi and its corresponding control treatment at the same time were significant at *p* < 0.05. An asterisk (^∗∗^) indicates that the differences were significant at *p* < 0.01. CK represents no-plant soil. Seedlings represents rhizosphere soil from sanqi.

FIGURE S9 Comparison of Bacteroidetes abundance differences between rhizosphere and no-plant soil at the genus level. The values represent the means ± SE. An asterisk (^∗^) indicates that the differences between sanqi and its corresponding control treatment at the same time were significant at *p* < 0.05. An asterisk (^∗∗^) indicates that the differences were significant at *p* < 0.01. CK represents no-plant soil. Seedlings represents rhizosphere soil from sanqi.

FIGURE S10 Comparison of Proteobacteria abundance differences between rhizosphere and no-plant soil at the genus level. The values represent the means ± SE. An asterisk (^∗^) indicates that the differences between sanqi and its corresponding control treatment at the same time were significant at *p* < 0.05. An asterisk (^∗∗^) indicates that the differences were significant at *p* < 0.01. CK represents no-plant soil. Seedlings represents rhizosphere soil from sanqi.

FIGURE S11 Comparison of Verrucomicrobia abundance differences between rhizosphere and no-plant soil at the genus level. The values represent the means ± SE. An asterisk (^∗^) indicates that the differences between sanqi and its corresponding control treatment at the same time were significant at *p* < 0.05. An asterisk (^∗∗^) indicates that the differences were significant at *p* < 0.01. CK represents no-plant soil. Seedlings represents rhizosphere soil from sanqi.

TABLE S1 Processed sample data information to analyze the fungal community.

TABLE S2 Processed sample data information to analyze the bacterial community.

TABLE S3 Antagonistic effect of *Trichoderma* spp. against the soil-borne pathogens of sanqi roots.

TABLE S4 Antagonistic effect of bacteria isolates against the soil-borne pathogens of sanqi roots.

TABLE S5 Effect of antagonistic bacteria on growth. + indicates growth on NA, resistant saponin medium or nitrogen-fixing medium; phosphorus or potassium-releasing circles on medium; − indicates opposite result.
