# Supplementary figures and images for: Negative Plant-Soil Feedback Driven by Re-assemblage of the Rhizosphere Microbiome With the Growth of Panax notoginseng
Source: Front Microbiol. 2019 Jul 26;10:1597. doi: 10.3389/fmicb.2019.01597 (PMC6676394; doi:10.3389/fmicb.2019.01597)

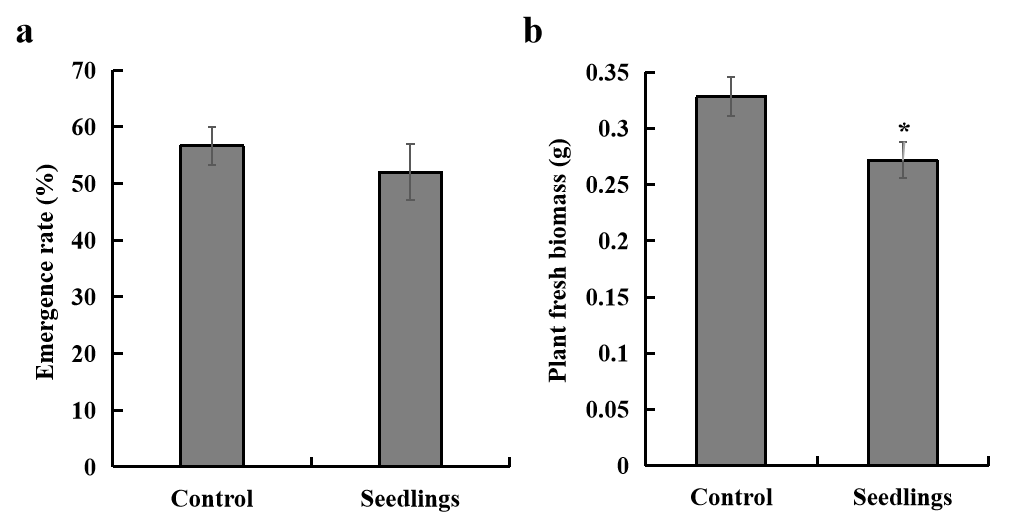

Supplement: FIGURE S1 — Seed germination (a) and plant fresh biomass (b) of per sanqi in the bulk soil without or with sanqi growth for 90 days in pots. Control represents the bulk soil without sanqi growth, seedlings represents the bulk soil with sanqi growth for 90 days in pots. The values represent the means ± SE. An asterisk (∗) indicates significant differences between different treatments (p < 0.05; n = 5). [file Image_1.TIF]

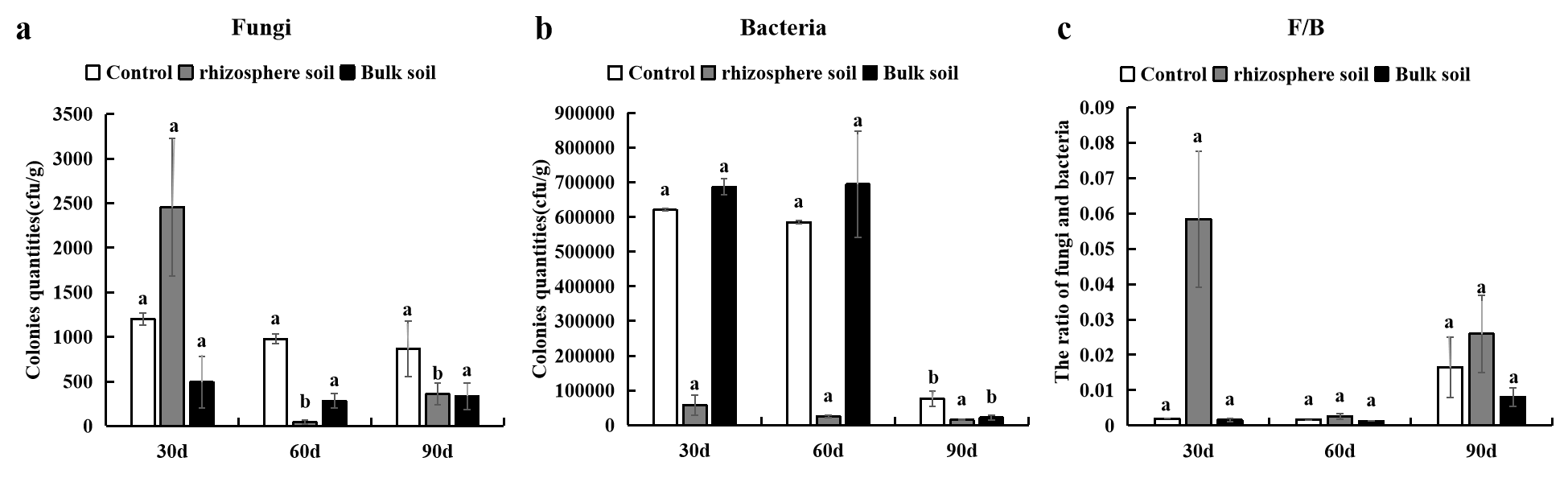

Supplement: FIGURE S2 — The influence of seedlings on culturable fungi (a) and bacteria (b) and the fungal to bacterial ratio (F/B) (c) in the rhizosphere and bulk soil. Control represents colony quantities or the ratio of fungi to bacteria in the soil without seedlings. Bulk soil represents colony quantities or the ratio of fungi to bacteria in the soil after removed seedlings. The values represent the means ± SE. Data in the homochromatic column with different lowercase letters indicate significant differences between different treatment durations (p < 0.05; n = 3). [file Image_2.tif]

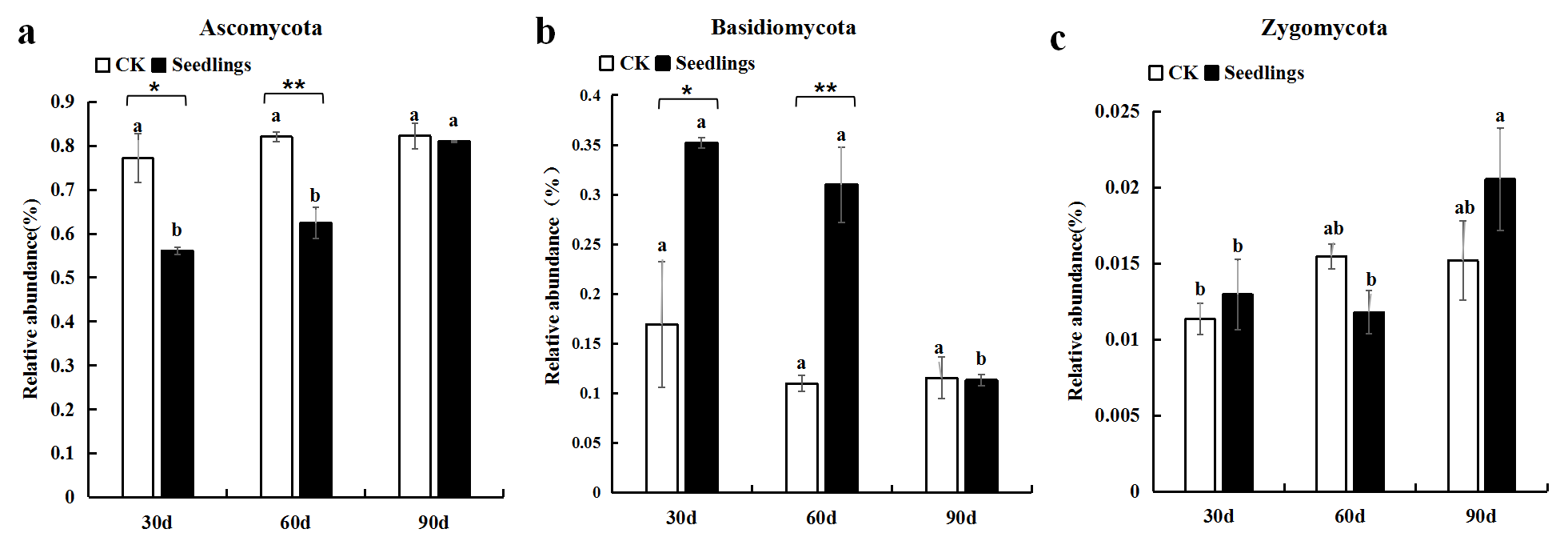

Supplement: FIGURE S3 — Effect of Panax notoginseng seedlings on the relative abundance of Ascomycota (a), Basidiomycota (b) and Zygomycota (c). The values represent the means ± SE. An asterisk (∗) indicates that the differences between seedlings and their corresponding control treatment at the same time were significant at p < 0.05. An asterisk (∗∗) indicates that the differences between seedlings and their corresponding control treatment at the same time were significant at p < 0.01. CK represents no-plant soil. Seedlings represents rhizosphere soil from sanqi. [file Image_3.TIF]

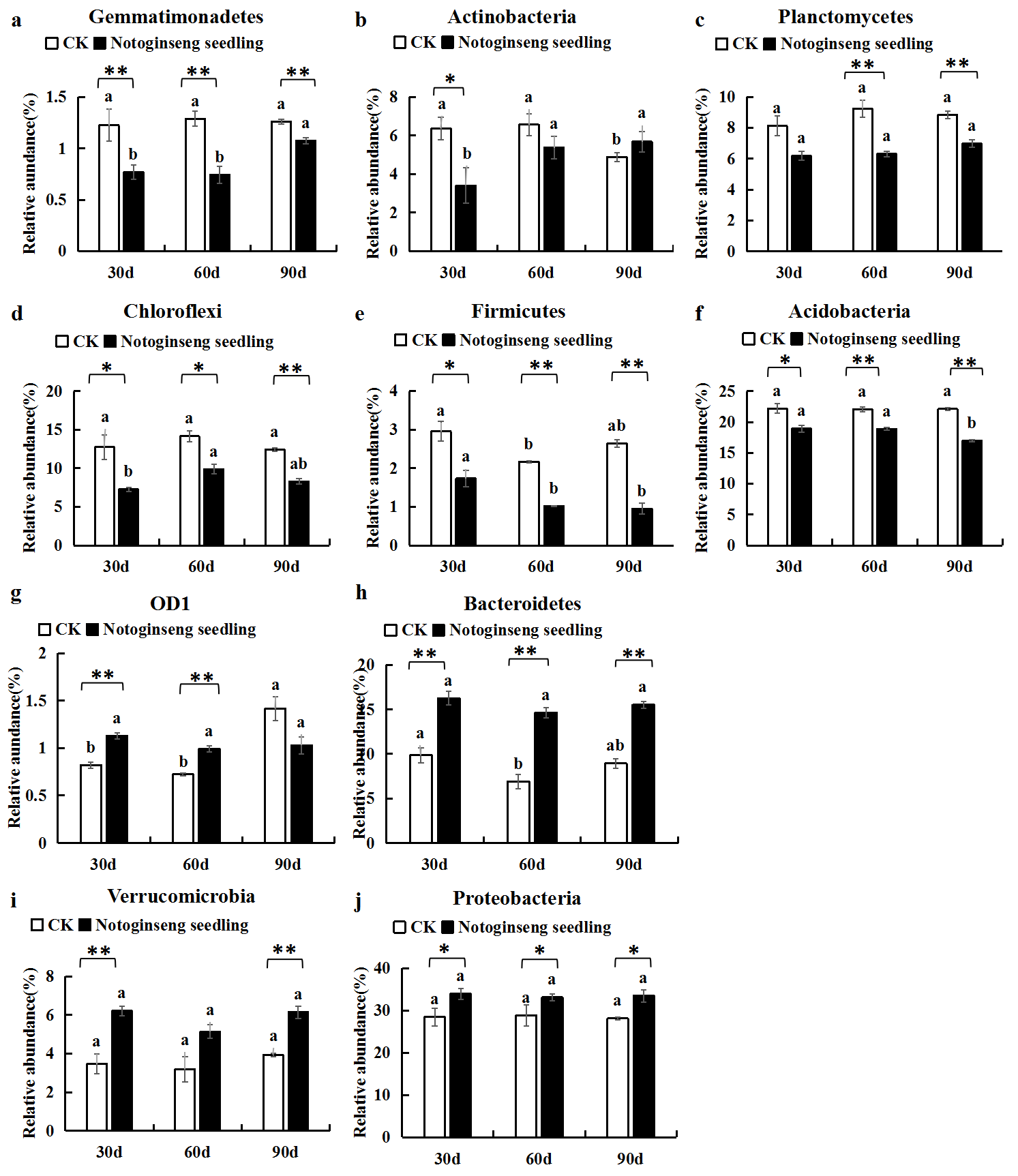

Supplement: FIGURE S4 — Effect of Panax notoginseng seedlings on the relative abundance of the dominant bacteria at the phylum level. a–j indicate that relative abundances of Gemmatimonadtes (a), Actinobacteria (b), Planctomycetes (c), Chloroflexi (d), Firmicutes (e) and Acidobacteria (f) were reduced and abundances of OD1 (g), Bacteroidetes (h), Verrucomicrobia (i) and Proteobacteria (j) were increased after planting sanqi, respectively. The values represent the means ± SE. An asterisk (∗) indicates that the differences between sanqi and its corresponding control treatment at the same time were significant at p < 0.05. An asterisk (∗∗) indicates that the differences were significant at p < 0.01. CK represents no-plant soil. Notoginseng seedling represents rhizosphere soil from sanqi. [file Image_4.TIF]

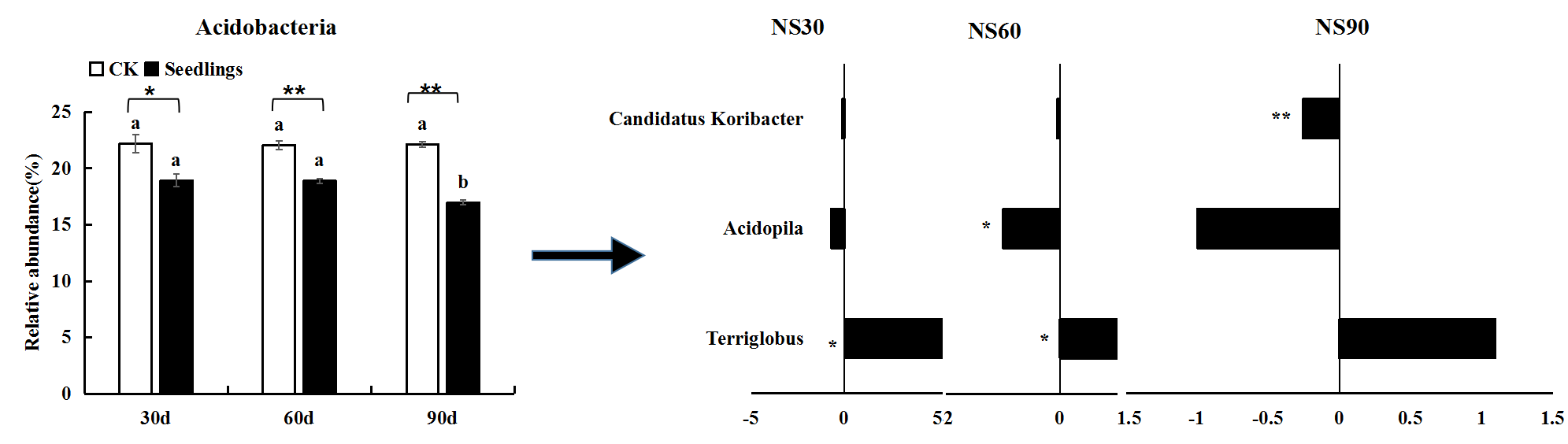

Supplement: FIGURE S5 — Comparison of Acidobacteria abundance differences between rhizosphere and no-plant soil at the genus level. The values represent the means ± SE. An asterisk (∗) indicates that the differences between sanqi and its corresponding control treatment at the same time were significant at p < 0.05. An asterisk (∗∗) indicates that the differences were significant at p < 0.01. CK represents no-plant soil. Seedlings represents rhizosphere soil from sanqi. [file Image_5.TIF]

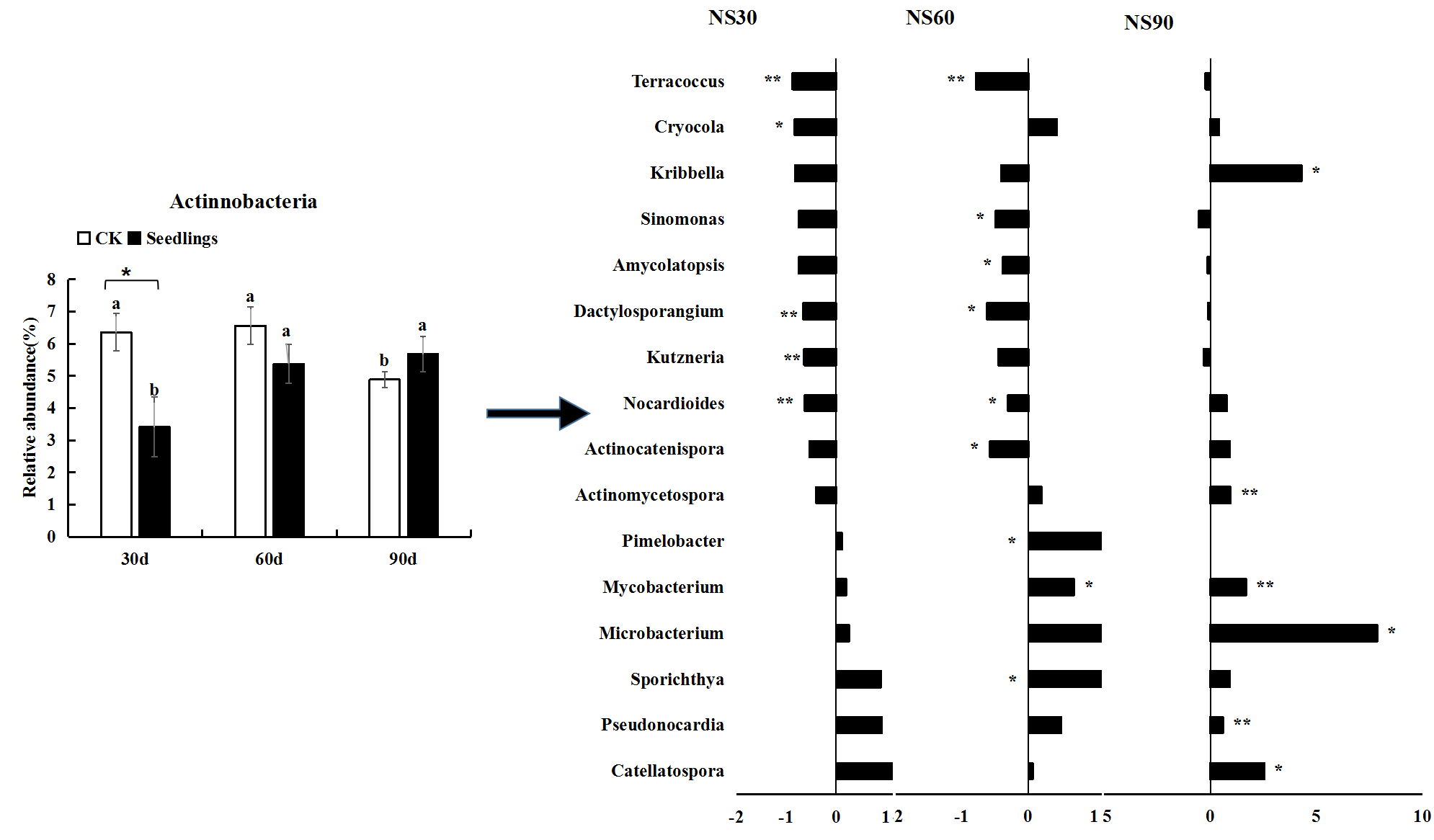

Supplement: FIGURE S6 — Comparison of Actinobacteria abundance differences between rhizosphere and no-plant soil at the genus level. The values represent the means ± SE. An asterisk (∗) indicates that the differences between sanqi and its corresponding control treatment at the same time were significant at p < 0.05. An asterisk (∗∗) indicates that the differences were significant at p < 0.01. CK represents no-plant soil. Seedlings represents rhizosphere soil from sanqi. [file Image_6.TIF]

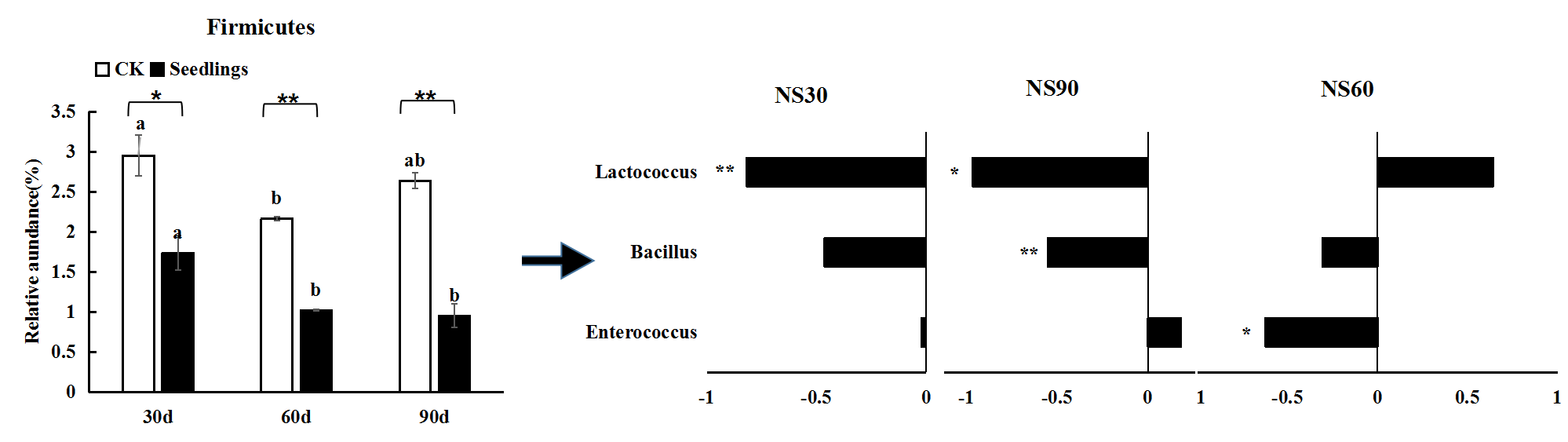

Supplement: FIGURE S7 — Comparison of Firmicutes abundance differences between rhizosphere and no-plant soil at the genus level. The values represent the means ± SE. An asterisk (∗) indicates that the differences between sanqi and its corresponding control treatment at the same time were significant at p < 0.05. An asterisk (∗∗) indicates that the differences were significant at p < 0.01. CK represents no-plant soil. Seedlings represents rhizosphere soil from sanqi. [file Image_7.TIF]

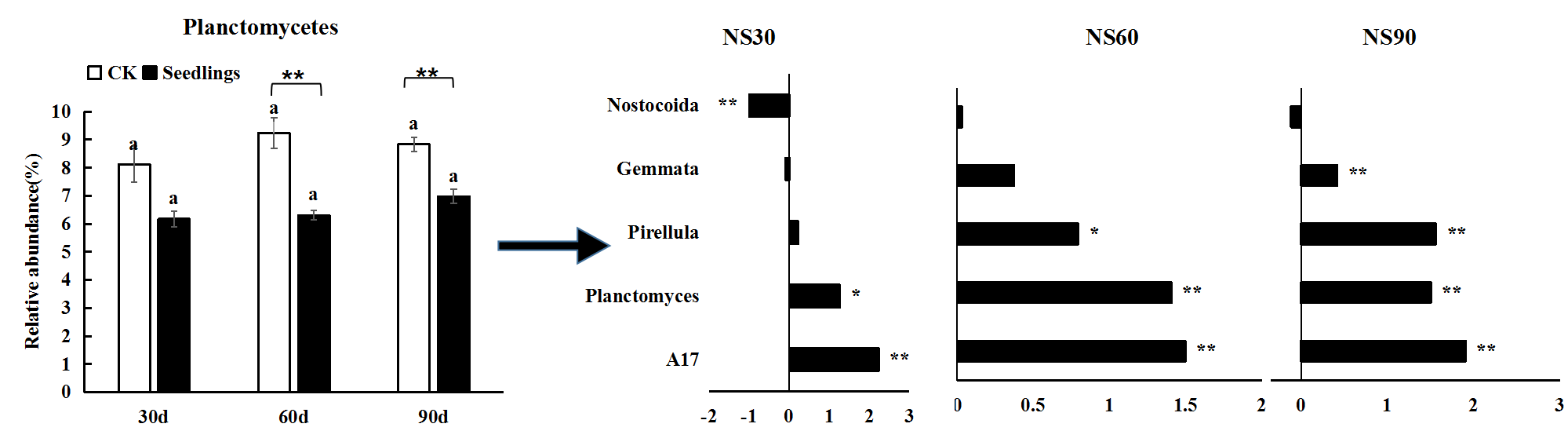

Supplement: FIGURE S8 — Comparison of Planctomycetes abundance differences between rhizosphere and no-plant soil at the genus level. The values represent the means ± SE. An asterisk (∗) indicates that the differences between sanqi and its corresponding control treatment at the same time were significant at p < 0.05. An asterisk (∗∗) indicates that the differences were significant at p < 0.01. CK represents no-plant soil. Seedlings represents rhizosphere soil from sanqi. [file Image_8.TIF]

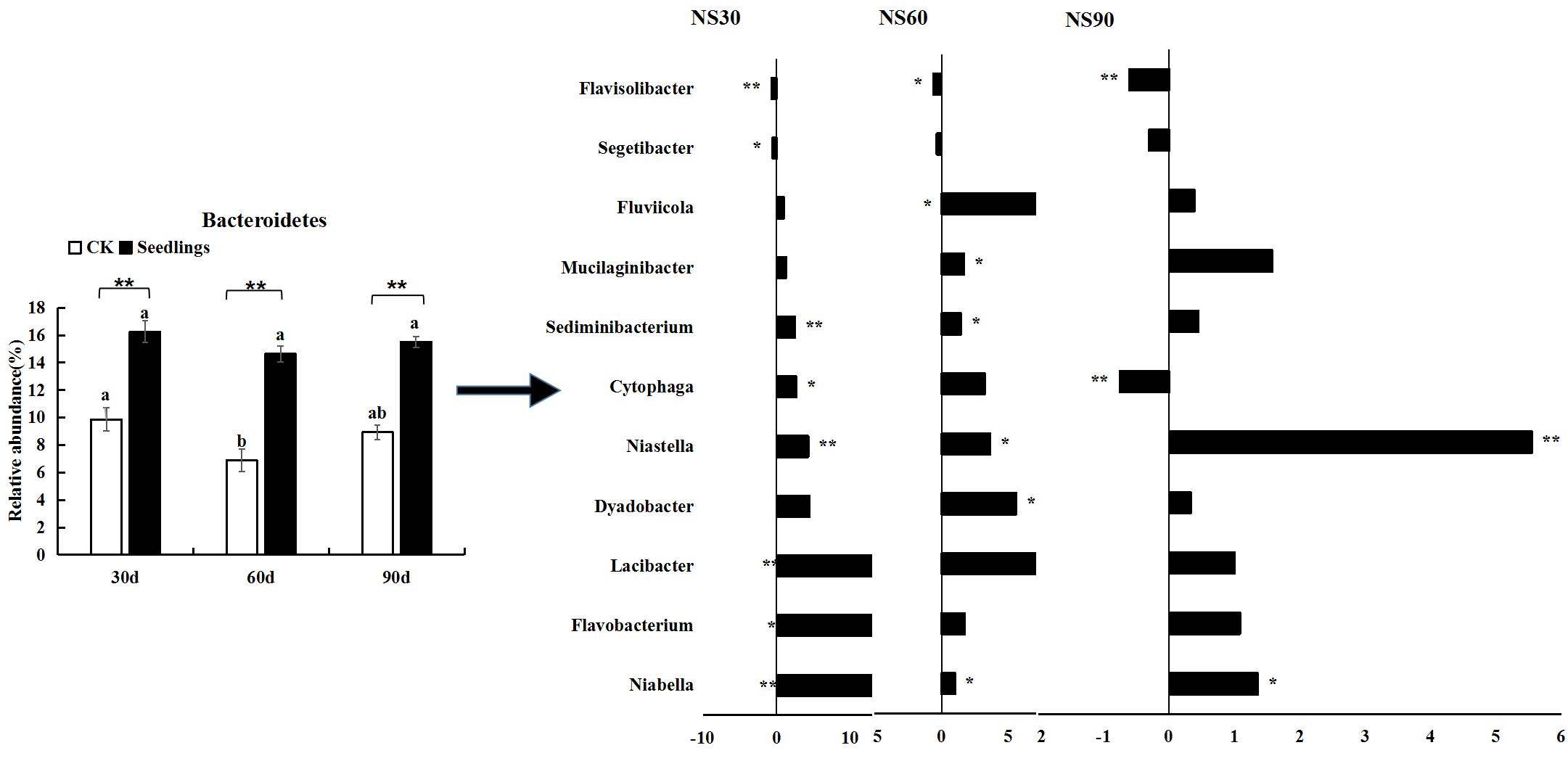

Supplement: FIGURE S9 — Comparison of Bacteroidetes abundance differences between rhizosphere and no-plant soil at the genus level. The values represent the means ± SE. An asterisk (∗) indicates that the differences between sanqi and its corresponding control treatment at the same time were significant at p < 0.05. An asterisk (∗∗) indicates that the differences were significant at p < 0.01. CK represents no-plant soil. Seedlings represents rhizosphere soil from sanqi. [file Image_9.TIF]

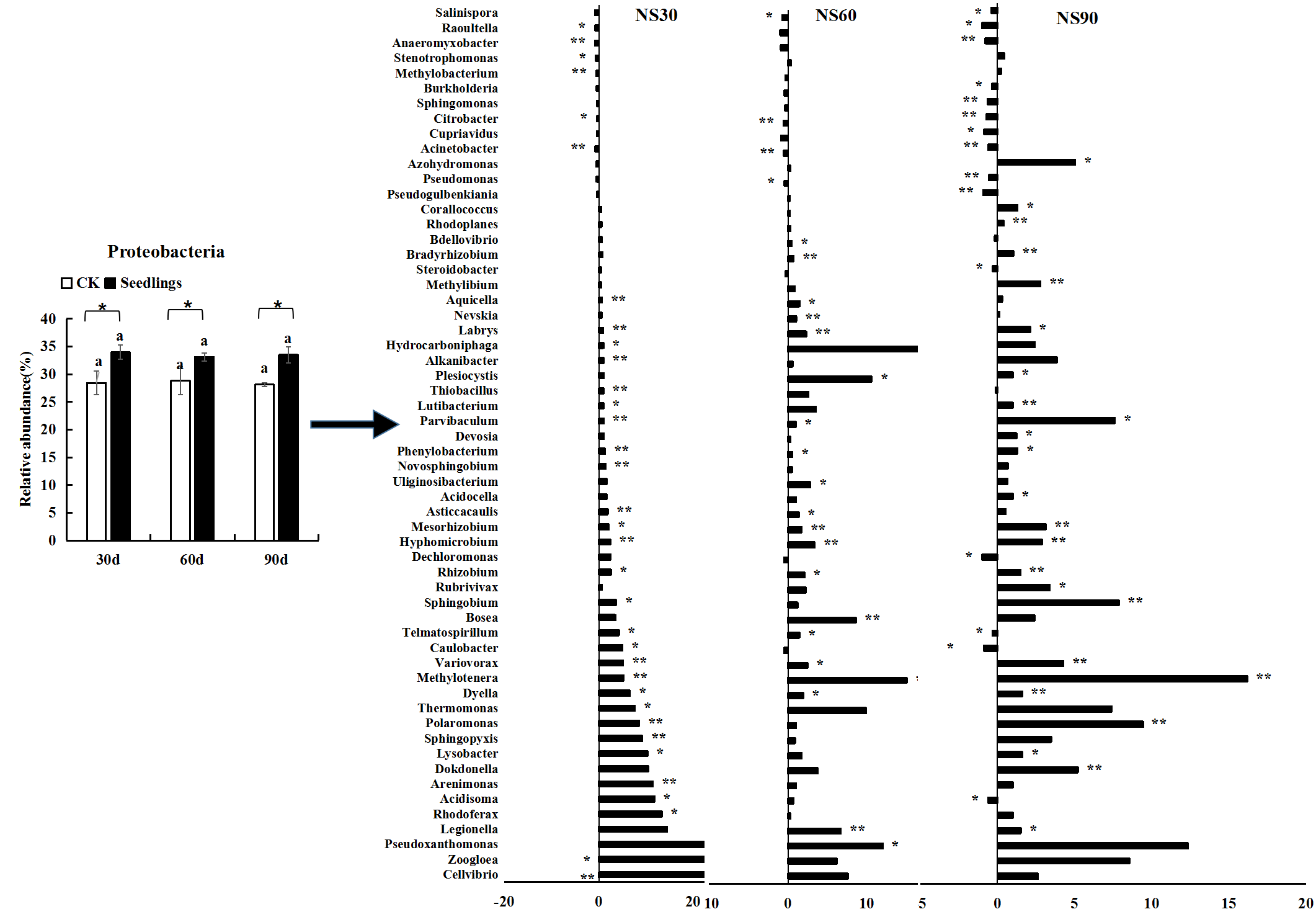

Supplement: FIGURE S10 — Comparison of Proteobacteria abundance differences between rhizosphere and no-plant soil at the genus level. The values represent the means ± SE. An asterisk (∗) indicates that the differences between sanqi and its corresponding control treatment at the same time were significant at p < 0.05. An asterisk (∗∗) indicates that the differences were significant at p < 0.01. CK represents no-plant soil. Seedlings represents rhizosphere soil from sanqi. [file Image_10.TIF]

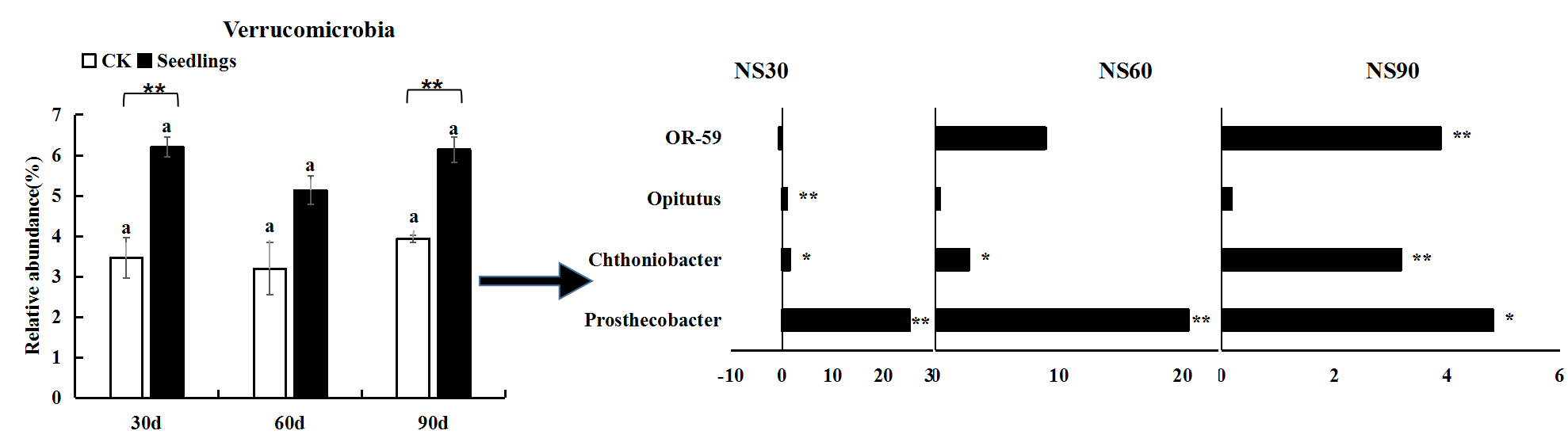

Supplement: FIGURE S11 — Comparison of Verrucomicrobia abundance differences between rhizosphere and no-plant soil at the genus level. The values represent the means ± SE. An asterisk (∗) indicates that the differences between sanqi and its corresponding control treatment at the same time were significant at p < 0.05. An asterisk (∗∗) indicates that the differences were significant at p < 0.01. CK represents no-plant soil. Seedlings represents rhizosphere soil from sanqi. [file Image_11.TIF]
