# Supplementary material for: Negative Plant-Soil Feedback Driven by Re-assemblage of the Rhizosphere Microbiome With the Growth of Panax notoginseng
Source: Front Microbiol. 2019 Jul 26;10:1597. doi: 10.3389/fmicb.2019.01597 (PMC6676394; doi:10.3389/fmicb.2019.01597)
Supplement: TABLE S1 — Processed sample data information to analyze the fungal community. [file Table_1.DOC]

Table S1 Processed sample data information to analyze fungal community

| Sample ID | Raw Reads | Clean Reads | AvgLen | Number of sequences | Number of OTUs |
| --- | --- | --- | --- | --- | --- |
| SCK.1 | 138015 | 135682 | 256.8 | 122596 | 1153 |
| SCK.2 | 31341 | 30681 | 257.43 | 23839 | 1120 |
| SCK.3 | 58490 | 57603 | 258.84 | 52283 | 1045 |
| SCK.4 | 68886 | 67848 | 258 | 60059 | 960 |
| SCK.5 | 65432 | 64270 | 258.27 | 57644 | 1071 |
| SCK.6 | 45111 | 44236 | 259.95 | 30507 | 1172 |
| SCK.7 | 60528 | 59383 | 258.65 | 51331 | 1064 |
| SCK.8 | 53645 | 52740 | 260.07 | 48139 | 964 |
| SCK.9 | 34143 | 33655 | 255.48 | 28068 | 869 |
| NS.1 | 107694 | 105799 | 256 | 93856 | 1346 |
| NS.2 | 88077 | 86678 | 259.94 | 80061 | 791 |
| NS.3 | 64002 | 62802 | 257.05 | 55834 | 977 |
| NS.4 | 58888 | 57717 | 263.33 | 53962 | 1016 |
| NS.5 | 70175 | 68922 | 258.68 | 63935 | 989 |
| NS.6 | 74924 | 73430 | 262.81 | 65497 | 795 |
| NS.7 | 63413 | 62427 | 258.91 | 54677 | 960 |
| NS.8 | 51753 | 50726 | 260.39 | 44005 | 943 |
| NS.9 | 46070 | 45313 | 255.58 | 37865 | 642 |
| Sum | 1180587 | 1159912 |  | 1024158 | 17877 |
