# Supplementary material for: Negative Plant-Soil Feedback Driven by Re-assemblage of the Rhizosphere Microbiome With the Growth of Panax notoginseng
Source: Front Microbiol. 2019 Jul 26;10:1597. doi: 10.3389/fmicb.2019.01597 (PMC6676394; doi:10.3389/fmicb.2019.01597)
Supplement: TABLE S2 — Processed sample data information to analyze the bacterial community. [file Table_2.DOC]

Table S2 Processed sample data information to analyze bacterial community

| Sample ID | Raw Reads | Clean Reads | AvgLen | Number of sequences | Number of OTUs |
| --- | --- | --- | --- | --- | --- |
| SCK.1 | 123941 | 114261 | 373.24 | 37910 | 3049 |
| SCK.2 | 198985 | 184398 | 373.17 | 63579 | 3781 |
| SCK.3 | 105666 | 95998 | 373.21 | 35219 | 3146 |
| SCK.4 | 112173 | 103056 | 373.23 | 38015 | 3191 |
| SCK.5 | 120101 | 110018 | 373.21 | 43529 | 3588 |
| SCK.6 | 152471 | 138386 | 373.44 | 50951 | 3769 |
| SCK.7 | 131459 | 120618 | 373.16 | 46190 | 3792 |
| SCK.8 | 149456 | 137395 | 373.19 | 51097 | 3760 |
| SCK.9 | 142932 | 131039 | 373.2 | 50394 | 3863 |
| NS.1 | 125571 | 115870 | 372.88 | 37842 | 2779 |
| NS.2 | 141704 | 128423 | 372.83 | 53588 | 3321 |
| NS.3 | 115285 | 105679 | 372.91 | 36465 | 2639 |
| NS.4 | 105975 | 97737 | 372.99 | 34934 | 2875 |
| NS.5 | 141491 | 130333 | 372.99 | 46226 | 3063 |
| NS.6 | 186526 | 169074 | 372.94 | 55725 | 2903 |
| NS.7 | 156642 | 143078 | 372.94 | 48651 | 3253 |
| NS.8 | 163136 | 148952 | 372.96 | 47244 | 3386 |
| NS.9 | 160993 | 146502 | 372.99 | 49806 | 3404 |
| Sum | 2534507 | 2320817 |  | 827365 | 59562 |
