# Supplementary material for: Negative Plant-Soil Feedback Driven by Re-assemblage of the Rhizosphere Microbiome With the Growth of Panax notoginseng
Source: Front Microbiol. 2019 Jul 26;10:1597. doi: 10.3389/fmicb.2019.01597 (PMC6676394; doi:10.3389/fmicb.2019.01597)
Supplement: TABLE S3 — Antagonistic effect of Trichoderma spp. against the soil-borne pathogens of sanqi roots. [file Table_3.DOC]

Table S3 Antagonistic effect of *Trichoderma* spp. againstthe soil-borne pathogens of sanqi roots

| Numer | Inhibiting rate (%) | | |
| --- | --- | --- | --- |
|  | *Fusarium solani* | *Fusarium oxysporum* | *Monographella cucumerina* |
| 3NG-1 | 82.76±0.50 | 66.28±1.33 | 89.44±0.32 |
| 50FN7 | 54.02±1.89 | 40.70±3.00 | 71.11±4.71 |
| 50FN2 | 70.11±2.46 | 51.16±1.45 | 89.44±0.32 |
| 2ZG-3 | 83.91±2.45 | 83.72±2.00 | 89.44±0.32 |
| 50FZ1 | 83.91±2.45 | 53.49±1.03 | 57.50±5.30 |
| 2Z2-2-2 | 91.95±1.50 | 79.07±1.64 | 89.44±0.32 |
