# Supplementary material for: Negative Plant-Soil Feedback Driven by Re-assemblage of the Rhizosphere Microbiome With the Growth of Panax notoginseng
Source: Front Microbiol. 2019 Jul 26;10:1597. doi: 10.3389/fmicb.2019.01597 (PMC6676394; doi:10.3389/fmicb.2019.01597)
Supplement: TABLE S4 — Antagonistic effect of bacteria isolates against the soil-borne pathogens of sanqi roots. [file Table_4.DOC]

Table S4 Antagonistic effect of bacteria isolates against the soil-borne pathogens of sanqi roots

| Phylum | Number | Antibacterial rate(%) | | |
| --- | --- | --- | --- | --- |
|  |  | *Fusarium solani* | *Fusarium oxysporum* | *Monographella cucumerina* |
| *Pseudomonas* | X-2-17 | 72.08±1.20 | 70.16±2.81 | 3.33±1.92 |
|  | X-2-16 | 74.49±0.81 | 71.48±2.15 | 5.00±3.19 |
|  | 200FZ5 | 26.69±3.45 | 41.27±5.72 | 46.43±2.47 |
|  | 4-8 | 73.61±0.18 | 72.86±1.80 | 19.05±1.93 |
|  | X-2-5 | 71.49±0.73 | 73.36±2.17 | 13.81±2.74 |
|  | S-16-2 | 75.89±0.81 | 75.5±1.84 | 3.33±1.92 |
|  | 200FZ4 | 43.54±7.44 | 21.43±3.64 | 3.45±2.00 |
|  | 2NCK-2 | 40.82±4.89 | 47.35±9.79 | 56.79±2.36 |
|  | 1N3 | 33.76±3.97 | 42.06±6.78 | 18.69±4.83 |
| *Acinetobacter* | 3NCK-1 | 60.63±3.32 | 65.34±3.44 | 5.00±3.19 |
|  | 1Z13 | 62.02±2.00 | 63.23±1.74 | 22.26±2.93 |
| *Burkholderia* | 200FZ1 | 39.49±1.99 | 34.66±6.49 | 58.57±0.82 |
|  | 1Z4 | 25.3±2.15 | 34.66±4.40 | 74.05±2.03 |
| *Bacillus* | CKZ7 | 62.02±2.00 | 59.52±5.00 | 68.93±2.09 |
|  | 200FZ3-2 | 61.90±2.94 | 49.47±5.50 | 65.48±0.69 |
|  | 1Z10 | 49.28±2.51 | 55.03±1.40 | 51.79±2.25 |
|  | 1ZG | 63.29±4.15 | 58.20±1.91 | 60.24±2.25 |
|  | 1Z | 60.51±3.12 | 56.61±2.80 | 50.00±1.36 |
|  | 3NCK-3 | 54.95±0.78 | 51.59±0.79 | 63.81±1.39 |
|  | 2Z2 | 68.90±4.19 | 71.69±0.27 | 64.23±2.52 |
|  | 1NG-6 | 60.63±3.33 | 69.84±3.18 | 55.12±2.19 |
|  | XN1 | 61.96±7.23 | 52.91±4.23 | 55.00±2.89 |
