# Supplementary material for: Negative Plant-Soil Feedback Driven by Re-assemblage of the Rhizosphere Microbiome With the Growth of Panax notoginseng
Source: Front Microbiol. 2019 Jul 26;10:1597. doi: 10.3389/fmicb.2019.01597 (PMC6676394; doi:10.3389/fmicb.2019.01597)
Supplement: TABLE S5 — Effect of antagonistic bacteria on growth. + indicates growth on NA, resistant saponin medium or nitrogen-fixing medium; phosphorus or potassium-releasing circles on medium; − indicates opposite result. [file Table_5.DOC]

Table S5 Effect of antagonistic bacteria on growth

| Number | NA | N-fixing | Phosphorus -releasing circles | Potassium-releasing circles |
| --- | --- | --- | --- | --- |
| X-2-17 | + | + | + | + |
| X-2-16 | + | + | + | + |
| 200FZ5 | + | + | + | + |
| 4-8 | + | + | + | + |
| X-2-5 | + | + | + | + |
| S-16-2 | + | + | + | + |
| 200FZ4 | + | + | + | + |
| 2NCK-2 | + | - | - | - |
| 1N3 | + | + | - | + |
| 3NCK-1 | + | + | - | - |
| 1Z13 | + | + | + | - |
| 200FZ1 | + | - | + | - |
| 1Z4 | + | + | + | + |
| CKZ7 | + | + | + | + |
| 200FZ3-2 | + | - | + | - |
| 1Z10 | + | + | + | + |
| 1ZG | + | + | - | + |
| 1Z | + | + | + | - |
| 3NCK-3 | + | - | + | - |
| 2Z2 | + | + | + | + |
| 1NG-6 | + | + | + | + |
| XN1 | + | + | - | + |

Note: + indicates growth on NA, resistant saponin medium or nitrogen fixing medium; Phosphorus or potassium-releasing circles on medium; - indicates opposite result.
